# Vitamin D Status and Supplementation and the Functional Outcomes of Human Musculoskeletal Tissues: A Stratified Systematic Review

**DOI:** 10.1002/hsr2.72407

**Published:** 2026-05-05

**Authors:** Mohammad Soltani, Ali Fatahi, Fatemeh Heidari

**Affiliations:** ^1^ Department of physical education and sport science CT.C, Islamic Azad University Tehran Iran; ^2^ Department of Anatomical Sciences, Cellular and Molecular Research Center Qom University of Medical Sciences Qom Iran

**Keywords:** bone, function, muscle, supplement, tendon, vitamin D

## Abstract

**Background and Aim:**

This systematic review examined associations between vitamin D status and/or supplementation and functional outcomes (e.g., strength, performance, falls), with secondary reporting of biomechanical endpoints of human muscle, tendon, and bone.

**Methods:**

Following PRISMA guidelines, we reviewed human studies published from 1997 to 2024 that evaluated vitamin D supplementation/intake or serum 25‐hydroxyvitamin D (25[OH]D) status in relation to functional outcomes and reported at least one biomechanical endpoint (e.g., strength‐related properties, stiffness/tensile characteristics, bone mineral density [BMD], tendon outcomes). Eligible studies were English or Persian, conducted in humans, and included a vitamin D exposure and functional outcomes with biomechanical reporting. We excluded non‐human and in vitro studies, conference abstracts without peer‐reviewed full text, reviews, and studies lacking either vitamin D exposure or relevant functional/biomechanical outcomes.

**Results:**

Across eligible studies, higher vitamin D status and/or supplementation was generally associated with better muscle‐related functional outcomes in older or clinical populations, particularly when baseline deficiency was present. Findings in athletic populations were mixed, with many studies showing null or inconsistent effects on strength and performance. Evidence suggested potential benefits for tendon repair in select contexts, and fracture risk reduction in specific populations; however, effects varied by baseline vitamin D status, participant characteristics, and outcome definitions. Large contemporary trials in broadly vitamin D–replete adults commonly reported no benefit for BMD or fracture outcomes.

**Conclusion:**

Vitamin D supplementation appears most beneficial in older/clinical groups with deficiency, with improvements observed in selected functional outcomes (notably balance/falls). In broadly replete adults, evidence does not support meaningful benefits for BMD or fractures, and in elite athletes, performance effects are inconsistent and often null. Overall, findings support correction of deficiency rather than universal supplementation for performance enhancement.

## 1 Introduction

Dietary supplements, defined as nutrients or compounds ingested to enhance health or performance beyond habitual diets [1], are widely used by athletes and the general population [2, 3]. Dietary supplements play a multifaceted role in optimizing an athlete's performance strategy. They support general health by ensuring sufficient intake of essential nutrients, correct micronutrient deficiencies, and provide energy and macronutrients that may be difficult to acquire exclusively through diet. Athletes frequently employ supplements to enhance performance directly, while also leveraging indirect benefits, such as facilitating rigorous training, optimizing body composition, alleviating musculoskeletal pain, accelerating recovery from injuries, and enhancing psychological well‐being [1, 4, 5]. Supplement use has surged significantly over the past two decades and has increasingly attracted the attention of consumers [6, 7, 8]. At the same time, the use of advanced scientific methods to explore dietary supplements has grown considerably. The articles in this special issue highlight progress in understanding nutrients like vitamin D, iron, omega‐3s, and iodine. However, research into botanicals and non‐nutrient compounds such as glucosamine, MSM, and coenzyme Q10 remains more challenging [9, 10]. Dietary supplements encompass a diverse array of constituents, including carbohydrates, proteins, lipids, minerals, vitamins, herbal compounds, enzymes, metabolic intermediates (e.g., specific amino acids), and various plant‐ or food‐derived extracts. These supplements can be categorized into three distinct groups based on their scientific validation. The first group includes supplements supported by a robust theoretical foundation and extensive empirical evidence confirming their efficacy and safety. The second group comprises supplements with a plausible scientific rationale but inconsistent evidence regarding their effectiveness, necessitating further research. The third group consists of supplements lacking credible scientific support, with studies consistently demonstrating inefficacy and potential health risks. This classification highlights the critical need for rigorous scientific scrutiny to establish the safety and efficacy of nutritional supplements [11].

Vitamin D, a prohormone, regulates calcium and phosphate metabolism, supporting bone growth, skeletal health, and muscle function [12]. Its anti‐inflammatory properties and role in reducing fracture risk, muscle weakness, and tendinopathy are well‐documented [13, 14]. The active form of vitamin D, 1,25‐dihydroxyvitamin D₃, helps regulate immune responses by decreasing pro‐inflammatory cytokines like IL‐6, TNF‐α, and IFN‐γ, while boosting the anti‐inflammatory cytokine IL‐10 in human blood cells infected with Mycobacterium tuberculosis [15]. Additionally, vitamin D helps control inflammation by influencing reactive oxygen species, cyclooxygenase activity, and the NF‐κB signaling pathway [16]. Vitamin D supplementation has been demonstrated to improve neuromuscular function, especially among older individuals [17]. It also serves a significant function in mitigating musculoskeletal injuries following exercise characterized by abnormal muscle contractions in athletes [18]. Additionally, it may have a crucial anti‐inflammatory role in conditions where inflammation is a contributing factor in the pathophysiological process [18]. Emerging evidence supports the critical involvement of vitamin D in the regenerative processes of skeletal muscle following injury [19]. Furthermore, vitamin D has demonstrated cytoprotective effects in injured tenocytes, suggesting its potential utility as a therapeutic agent in the treatment of tendinopathies [20]. It has been shown to enhance tenocyte proliferation and restore tendon‐specific markers through activation of the extracellular signal‐regulated kinase (ERK) and p38 mitogen‐activated protein kinase pathways [20]. These pathways underscore vitamin D's capacity to modulate immune responses and stimulate cellular repair. Additionally, sufficient levels of both vitamin D and calcium are essential for effective bone fracture healing [21], and professional athletes with vitamin D deficiency may face an elevated risk of fracture occurrence [22]. Scientific findings suggest that vitamin D significantly contributes to the tissue repair process by modulating cellular activities and immune system functions. In addition, maintaining sufficient levels of both calcium and vitamin D is critical for preserving bone strength, especially among athletes, where inadequate levels may result in weakened skeletal structure and a heightened risk of fractures [22].

Tissue biomechanical properties—stiffness, maximum force (Fmax), absorbed energy, and deformation—reflect their functional capacity [23]. Stiffness governs resistance to deformation, Fmax indicates ultimate strength, absorbed energy measures toughness, and deformation reflects elasticity [24]. The morphological quality of tissues, including cellular structure, composition, and organization of the extracellular matrix, and their molecular properties, is the main determinant of the biomechanical properties of tissues, and changes in this quality can lead to significant changes in the mechanical function of tissues [25, 26, 27].

However, while extensive research links vitamin D to musculoskeletal health, its tissue‐specific effects—particularly on intrinsic biomechanical properties of tendon, muscle, and bone—remain underexplored and inconsistently reported in human studies, with variable findings across populations. Therefore, this systematic review primarily synthesizes functional outcomes associated with vitamin D status or supplementation, and secondarily summarizes any reported biomechanical endpoints when available. Existing reviews commonly address (i) pediatric bone outcomes and clinical guidance during growth (e.g., bone density/fracture considerations), or (ii) athletic performance and injury risk in sport settings; however, these syntheses are often domain‐specific, do not consistently separate *functional/clinical outcomes* from *intrinsic biomechanical properties*, and rarely integrate evidence across muscle, tendon, and bone within a single framework. In addition, human studies reporting direct biomechanical endpoints (e.g., stiffness, tensile properties) remain sparse and heterogeneously measured, limiting inference about whether vitamin D influences intrinsic tissue mechanics versus downstream functional performance.

Accordingly, the present review is designed to clarify (1) which functional outcomes show the most consistent associations with vitamin D status or supplementation, (2) whether any biomechanical endpoints have been reported in humans and what they suggest, and (3) how findings differ by population and baseline vitamin D status. To avoid overgeneralization, we pre‐specify outcome‐domain tagging (biomechanical vs. functional vs. clinical) and interpret results within population strata (e.g., clinical/older, post‐operative, athletic cohorts), rather than extrapolating across settings.

## 2 Glossary of Terms

The distinction between biomechanics, function, and healing in human tissues such as muscle, tendon, and bone can be understood as follows: Throughout, we use “biomechanical” only for tissue mechanical properties, “functional” for performance tests, and “clinical” for events/symptoms; tables include a domain tag for each outcome.

### 2.1 Biomechanics

Biomechanics refers to the study of the mechanical properties and behavior of biological tissues under various forces and motions. It involves understanding how tissues like muscle, tendon, and bone respond to loads, transmit forces, and maintain structural integrity [28]. For example:

**Muscles** generate force and movement by contracting and pulling on tendons and bones, with their biomechanics involving force generation, length changes, and interaction with skeletal structures [29].

**Tendons** connect muscles to bones and transmit tensile forces to enable joint movement and stability. They exhibit viscoelastic properties, meaning their mechanical response depends on the rate of loading, allowing them to absorb energy at low strain rates and transmit force efficiently at high strain rates [30, 31].

**Bones** provide rigid support and resist compressive forces, with biomechanical properties tailored to withstand daily mechanical stresses while protecting organs and enabling movement [32].

Biomechanics integrates physics and engineering principles to describe tissue motion and force transmission, essential for understanding normal function and injury mechanisms [33].

### 2.2 Function

Function refers to the physiological role and performance of the tissue in the musculoskeletal system:

**Muscle** function involves generating contractile force to produce movement and stabilize joints [34].

**Tendon** function is primarily to transmit muscle‐generated forces to bones, enabling joint motion and preventing excessive displacement [30, 31].

**Bone** function includes providing structural support, facilitating movement through joints, and protecting internal organs [35].

Function depends on the tissue's biomechanical properties and its integration within the musculoskeletal system. For example, tendons maintain smooth mechanics during joint motion and prevent injury by limiting joint displacement beyond anatomical barriers [30].

### 2.3 Healing

Healing is the biological process by which tissues repair themselves after injury, restoring structure and function. Healing mechanisms differ among muscle, tendon, and bone due to their unique cellular composition, vascularity, and biomechanical environment:

**Muscle** healing involves regeneration of muscle fibers and restoration of contractile function, often supported by satellite cells and influenced by mechanical loading [34].

**Tendon** healing involves multiple cell types (fibroblasts, inflammatory cells, stem cells) that migrate and proliferate to repair damaged collagen matrix. Healing outcomes depend on tendon anatomy and physiology, with challenges such as scar tissue formation and adhesion impairing function. Mechanical stimulation plays a critical role in promoting cell proliferation, differentiation, and extracellular matrix remodeling during healing, but inappropriate loading can hinder repair [36, 37].

**Bone** healing is characterized by a well‐orchestrated process involving osteoblasts, osteoclasts, and osteocytes to restore mineralized matrix and mechanical strength. Bone has a richer blood supply than tendon, facilitating more robust healing [35].

At the tendon‐bone interface, healing is particularly complex due to the differing biomechanical and biochemical properties of tendon and bone. Mechanical signals sensed by cells via integrins and other mechanotransducers regulate healing by influencing cell behavior and matrix synthesis. Optimizing mechanical environments through controlled loading can enhance healing quality and functional recovery [30, 36, 37].

In conclusion, biomechanics describes the physical behavior of tissues under load; function refers to their physiological roles in movement and stability; and healing is the biological restoration process that depends on tissue‐specific cellular and mechanical factors. Understanding these distinctions is critical for improving clinical interventions and rehabilitation strategies in musculoskeletal injuries [31, 33]. Table 1 provide a distinction between the mentioned concepts including biomechanics, function, and healing in human different tissues as muscle, tendon, and bone.

**Table 1:** Distinction between biomechanics, function, and healing in human tissue (muscle, tendon, bone).

Aspect	Muscle	Tendon	Bone
Biomechanics	Force generation and contraction	Tensile load transmission; viscoelastic	Compressive strength and rigidity
Function	Movement and joint stabilization	Transmit muscle force to bone; joint stability	Structural support and protection
Healing	Fiber regeneration via satellite cells	Complex cell migration and matrix remodeling; sensitive to mechanical loading	Mineralized matrix restoration; rich vascularity

## 3 Methods

### 3.1 Study Design

This systematic review adhered to the Preferred Reporting Items for Systematic Reviews and Meta‐Analyses (PRISMA) statement [38, 39]. It evaluated randomized controlled trials (RCTs) and observational studies assessing vitamin D consumption and functional outcomes (primary); biomechanical endpoints (secondary, when reported) in human tissues.

### 3.2 Search Strategy

We searched PubMed (MEDLINE), Scopus, CINAHL, SportDiscus, and Google Scholar from 1 January 1997 to 31 March 2024 (date of last search: 31 March 2024). The strategy combined controlled vocabulary (e.g., MeSH where available) and free‐text keywords for (i) vitamin D exposure (“vitamin D”, cholecalciferol, ergocalciferol, calcifediol, “25‐hydroxyvitamin D”, “25(OH)D”), (ii) musculoskeletal tissues (muscle, tendon, bone), and (iii) outcomes (function*, performance, strength, power, biomechanics, stiffness, tensile, elasticity, “bone mineral density”, fracture healing), using Boolean operators and database‐specific field tags. Database‐specific, reproducible search strings for each platform (including limits/filters) are provided in Supplementary list. Searches were limited to English and Persian language records. To identify additional and grey literature records, we screened Google Scholar results and searched Persian databases (SID, IRANDOC, MAGIRAN, IranMedex, Medilib). We also performed backward reference‐list screening of all included studies and relevant reviews, and forward citation searching (Scopus/Google Scholar) to capture eligible articles not retrieved in the initial database searches. Only peer‐reviewed full‐text articles were eligible; conference abstracts, theses/dissertations, and non‐peer‐reviewed preprints were excluded. Full electronic search strategies (last search: 31 March 2024) is presented as below:

### 3.3 Pubmed (MEDLINE)

(“vitamin D” OR cholecalciferol OR ergocalciferol OR calcifediol OR “25‐hydroxyvitamin D” OR 25(OH)D AND (muscle OR tendon OR bone OR musculoskeletal AND (biomechanics OR stiffness [OR tensile OR strength OR performance OR “bone mineral density” OR fracture OR healing OR falls)

### 3.4 Scopus (TITLEABS‐KEY)

TITLE‐ABS‐KEY ((“Vitamin D” OR cholecalciferol OR ergocalciferol OR calcifediol OR “25‐hydroxyvitamin d” OR “25(OH)D”) AND (muscle OR tendon OR bone OR musculoskeletal) AND (biomechanics OR stiffness OR tensile OR strength OR performance OR “bone mineral density” OR fracture OR healing OR falls)).

### 3.5 CINAHL

(MH “Vitamin D + “OR TI (“vitamin D” OR cholecalciferol OR ergocalciferol OR calcifediol OR “25‐hydroxyvitamin d”) OR AB (“vitamin d” OR cholecalciferol OR ergocalciferol OR calcifediol OR “25‐hydroxyvitamin d”)) AND (TI/AB (muscle OR tendon OR bone OR musculoskeletal)) AND (TI/AB (biomechanics OR stiffness OR tensile OR strength OR performance OR “bone mineral density” OR fracture OR healing OR falls)).

- **SportDiscus** (same logic as CINAHL; adapt to its controlled vocabulary if used)
- **Google Scholar**

“Vitamin D” (muscle OR tendon OR bone) (strength OR performance OR biomechanics OR stiffness OR fracture OR healing OR falls).

### 3.6 Persian Databases

Provide Persian equivalents of: vitamin D/25(OH)D/muscle/tendon/bone/biomechanics/strength/function, and state any platform constraints (e.g., simple keyword search only).

To improve interpretability across studies using different assays and thresholds, we report authors' original 25(OH)D categories and additionally map values to commonly used clinical frameworks. Under the Institute of Medicine (IOM; now NASEM) approach, serum 25(OH)D concentrations of **> = 20 ng/mL (> = 50 nmol/L)** are considered sufficient for most individuals, while concentrations **< 12 ng/mL (< 30 nmol/L)** indicate increased risk of deficiency. The 2011 Endocrine Society guideline defined vitamin D deficiency as **< 20 ng/mL (< 50 nmol/L)** and insufficiency as **21–29 ng/mL (52.5–72.5 nmol/L)**; however, the Endocrine Society's 2024 guideline emphasizes that outcome‐specific thresholds for disease prevention remain uncertain and no longer endorses a universal target of 30 ng/mL. Given this variability, we avoid overinterpreting small between‐study differences around any single cut‐off and prioritize within‐study definitions for primary interpretation.

### 3.7 Eligibility Criteria

#### 3.7.1 Research Inclusion Criteria

We included human randomized controlled trials and observational studies (cohort, case‐control, cross‐sectional) that evaluated (a) vitamin D status (serum/plasma 25(OH)D) and/or (b) vitamin D supplementation (vitamin D2/D3 or calcifediol), and reported at least one prespecified outcome in muscle, tendon, or bone. Outcomes were operationalized a priori into domains: (1) Functional/Performance outcomes (e.g., muscle strength, power, jump/sprint performance, balance, gait, functional tests), (2) Clinical outcomes (e.g., injury incidence, pain, falls, return‐to‐sport/return‐to‐function, fracture or tendon healing outcomes), and (3) Biomechanical endpoints (intrinsic or structural properties such as stiffness, elasticity/modulus, tensile strength, load to failure, torque‐angle characteristics; and bone structural strength indices where directly reported). Studies were eligible in participants described as healthy or clinical/athletic populations; for “healthy” samples we required absence of conditions or medications known to materially affect vitamin D metabolism or musculoskeletal outcomes (e.g., chronic kidney/liver disease, malabsorption syndromes, endocrine/metabolic bone disorders, neuromuscular disease, long‐term glucocorticoid/antiepileptic therapy). If mixed populations were included, studies were retained only when outcomes for eligible participants were reported separately. Only peer‐reviewed studies published in reputable journals with direct relevance to human musculoskeletal tissues were included.

#### 3.7.2 Research Exclusion Criteria

Studies were excluded if they were non‐human or in‐vitro, involved primary conditions unrelated to musculoskeletal performance/healing (or conditions that confounded vitamin D metabolism/outcomes without separable data), incorporated pharmacologic or nutritional interventions beyond vitamin D that were not balanced across groups, targeted non‐relevant tissues, or consisted of conference abstracts without peer‐reviewed full‐text publications. Review articles were excluded (but their reference lists were screened for eligible primary studies). Following the screening process, 49 studies were included for analysis.

### 3.8 Risk of Bias Assessment

We excluded non‐human and in‐vitro studies from the main synthesis; mechanistic/preclinical evidence is summarized qualitatively to contextualize human findings. Risk of bias and methodological quality were assessed at the study level and used to weight the narrative synthesis. Randomized controlled trials (RCTs) were appraised using the PEDro scale (0–11). To enhance interpretability, PEDro items were mapped to common bias domains: selection bias (eligibility criteria, random allocation, allocation concealment, baseline comparability), performance bias (participant and therapist blinding), detection bias (assessor blinding), attrition bias (adequate follow‐up and intention‐to‐treat analysis), and reporting bias (between‐group comparisons and reporting of point estimates/variability). Consistent with established PEDro guidance, scores > = 7 were considered high quality.

Three reviewers independently screened studies and independently scored PEDro items for each eligible RCT; disagreements were resolved by discussion and, when needed, adjudication by a third reviewer. Potential publication bias and selective outcome reporting were considered by comparing outcomes described in Methods versus those reported in Results when protocols were unavailable.

### 3.9 Data Analysis

We anticipated substantial clinical and methodological heterogeneity across studies, including age ranges, 25(OH)D measurement techniques and reporting units, deficiency cut‐off thresholds, supplementation dose/form/duration, co‐interventions (e.g., calcium or exercise), and non‐comparable functional or biomechanical outcomes. Therefore, we planned a stratified narrative synthesis a priori by (i) age group (children < = 12 years, adolescents 13–18 years, and other adult/older cohorts when present), (ii) population context (athletes, general/clinical populations, and post‐operative cohorts), and (iii) outcome domain (Functional/Performance, Clinical, and Biomechanical endpoints). For each study, we extracted the 25(OH)D assay method (when reported), units, and author‐defined thresholds; where possible, values were harmonized to nmol/L (ng/mL x 2.496) and deficiency categories were mapped to commonly used cut‐offs (deficient < 50 nmol/L, insufficient 50–75 nmol/L, sufficient > = 75 nmol/L), while retaining author‐defined categories for sensitivity checks. Where sufficient data were available, we extracted or computed effect sizes (standardized mean differences for continuous outcomes; odds ratios or risk ratios for dichotomous outcomes) with 95% confidence intervals from reported summary statistics. Random‐effects meta‐analysis was considered; however, quantitative pooling was not performed because heterogeneity in interventions, baseline vitamin D status definitions, and outcome measures precluded meaningful synthesis. Instead, we summarize the direction and magnitude of effects within prespecified subgroups and highlight consistency across studies. Conclusions were weighted toward higher‐quality RCTs (PEDro > = 7), and sensitivity considerations included excluding lower‐quality trials and examining dose/form (D2 vs D3) when feasible. Three reviewers extracted data on study design, sample size, vitamin D dosage, duration, participant characteristics, and outcomes.

In addition to reporting PEDro total scores, we provide a brief domain‐oriented risk‐of‐bias summary across RCTs. Common strengths included randomization, baseline comparability, and complete reporting of between‐group effects; common limitations included limited feasibility of participant/therapist blinding and variable reporting of allocation concealment.

## 4 Results

Population‐ and age‐stratified narrative synthesis: Given heterogeneity in age ranges, 25(OH)D assays/thresholds, and outcome definitions, findings are organized by age group (children vs adolescents vs adult/older cohorts when included) and by population context (athletes, general/clinical, and post‐operative cohorts), with outcomes further grouped into Functional/Performance, Clinical, and Biomechanical domains.

**Figure 1:**
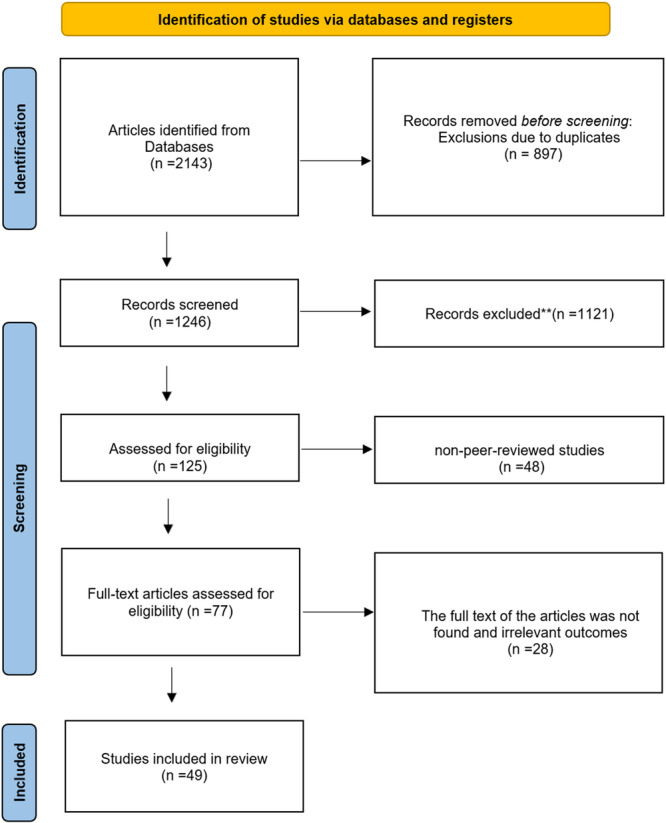
Screening procedure of Articles.

From 2143 initial articles, 1246 remained after deduplication, 125 were assessed for eligibility, and 49 met inclusion criteria (Figure 1). Key findings are summarized below and detailed in Tables 2, 3 and 4. For interpretability, we report findings stratified by population (older/clinical, post‐operative, athletes) and by outcome domain Functional/Clinical (with limited biomechanical endpoints).

**Table 2:** Summary of Key Studies on Vitamin D and Functional Outcomes of human skeletal muscles.

Author, year, ref	Title	Intervention	Outcome	Effect
Rebolledo et al. (2018) [40]	The Association of Vitamin D Status in Lower Extremity Muscle Strains and Core Muscle Injuries at the National Football League Combine	Observational, NFL combine	Lower muscle extremity strain risk with low 25[OH]D (*p* < 0.05)	**⇧**
Bauer et al. (2019) [41]	High prevalence of vitamin D insufficiency in professional handball athletes	Observational, NFL players	Increased risk of musculoskeletal injuries with insufficient vitamin D levels	**⇧**
Ong et al. (2024) [42]	Vitamin D as an intervention for improving quadriceps muscle strength in patients after anterior cruciate ligament reconstruction: study protocol for a randomized double‐blinded, placebo‐controlled clinical trial	4000 IU/day vitamin D3, 6 months	Improved strength recovery quadriceps strength post‐ACLR	**⇧**
Żebrowska et al. (2020) [43]	The effect of vitamin D supplementation on serum total 25(OH) levels and biochemical markers of skeletal muscles in runners	2000 IU/day vitamin D3, 8 weeks	Reducing the likelihood of skeletal muscle damage following eccentric exercise in athletes	**⇧**
Ceglia et al. (2013) [44]	A randomized study on the effect of vitamin D₃ supplementation on skeletal muscle morphology and vitamin D receptor concentration in older women	4000 IU/day vitamin D3, 4 months	Increased muscle fiber size by 10%	**⇧**
Visser et al. (2003) [45]	Low vitamin D and high parathyroid hormone levels as determinants of loss of muscle strength and muscle mass (sarcopenia): the Longitudinal Aging Study Amsterdam	Observational, sarcopenia	Reduced 25‐OHD levels are associated with a heightened risk of sarcopenia in elderly men and women and sarcopenia was defined as a loss of grip strength greater than 40%.	**⇧**
Bischoff et al. (2003) [46]	Effects of vitamin D and calcium supplementation on falls: a randomized controlled trial	1200 mg/day Calcium, 1200 mg/day vitamin D3, 12 weeks	49 percent reduction in falls following vitamin D supplementation in older women with vitamin D deficiency that likely due to improvements in muscle and bone function.	**⇧**
Dhesi et al. (2004) [17]	Vitamin D supplementation improves neuromuscular function in older people who fall	100,000 IU vitamin D2, single dose	Vitamin D significantly improve balance, reaction time, and overall physical function and have little effect on muscle strength. These improvements in neuromuscular function may help lowers the risk of falls and fractures.	**⇧**
Pfeifer et al. (2009) [47]	Effects of a long‐term vitamin D and calcium supplementation on falls and parameters of muscle function in community‐dwelling older individuals	800 IU/day vitamin D + calcium, 12 months	Taking both calcium and vitamin D together was more effective than calcium by itself in lowering fall rates and boosting muscle function among older.	**⇧**
Ceglia et al. (2010) [48]	Multi‐step immunofluorescent analysis of vitamin D receptor loci and myosin heavy chain isoforms in human skeletal muscle	Vitamin D3	An effective method for investigating potential associations between specific muscle fiber types and the expression levels of vitamin D receptors	**⇧**
Magalhães et al. (2024) [49]	Vitamin D Inadequacy and Its Relation to Body Fat and Muscle Mass in Adult Women of Childbearing Age	Vitamin D3	Positive correlation between 25(OH)D and muscle mass and a negative correlation with body fat.	**⇧**
Chen et al. (2022) [50]	Association of Preoperative Vitamin D Deficiency with Retear Rate and Early Pain After Arthroscopic Rotator Cuff Repair: A Retrospective Cohort Study	Observational, post‐RCR	Inadequate preoperative vitamin D levels were associated with a higher incidence of tendon rupture and increased pain after rotator cuff arthroscopy.	**⇧**
Lee et al. (2021) [51]	Prevalence of and Risk Factors for Hypovitaminosis D in Patients with Rotator Cuff Tears	Observational, rotator cuff tears	Hypovitaminosis D was found in 44.3% of patients with rotator cuff tears.	**⇧**
Harada et al. (2019) [52]	Preoperative Vitamin D Deficiency Is Associated with Higher Postoperative Complications in Arthroscopic Rotator Cuff Repair	Observational, pre‐RCR	A lack of vitamin D is associated with an increased risk of complications after arthroscopic rotator cuff repair and may be a changeable risk factor.	**⇧**
Oh et al. (2009) [53]	The level of vitamin D in the serum correlates with fatty degeneration of the muscles of the rotator cuff	Observational, pre‐RCR	Serum vitamin D levels are significantly inversely associated with fatty infiltration in rotator cuff muscles and positively correlated with isokinetic muscle torque.	**⇧**
Cancienne et al. (2019) [54]	Perioperative Serum 25‐Hydroxyvitamin D Levels Affect Revision Surgery Rates After Arthroscopic Rotator Cuff Repair	Observational, post‐RCR	This study found a statistically significant link between low serum 25‐hydroxyvitamin D levels and higher rates of revision rotator cuff surgery after initial arthroscopic repair.	**⇧**
Borges et al. (2020) [55]	Muscle degradation, vitamin D and systemic inflammation in hospitalized septic patients	Vitamin D3	In septic patients, Vitamin D levels were linked to improved muscle strength recovery during hospital stays.	**⇧**
Geusens et al. (1997) [56]	Quadriceps and grip strength are related to vitamin D receptor genotype in elderly nonobese women	Vitamin D3	A specific VDR gene variant is strongly linked to muscle strength in elderly non‐obese women.	**⇧**
De Rezende et al. (2020) [57]	The Relationship between Vitamin D Levels, Injury and Muscle Function in Adolescent Dancers	Vitamin D3	Serum 25‐hydroxyvitamin D levels show no correlation with muscle strength, while reduced strength is associated with higher injury risk	**⇔**
Dechsupa et al. (2023) [58]	Vitamin D Inadequacy Affects Skeletal Muscle Index and Physical Performance in Lumbar Disc Degeneration	Vitamin D3	Supplementing vitamin D may enhance muscle strength, physical function, and quality of life in patients with lumbar degenerative disease (LDD)	**⇧**
Javadian et al. (2017) [59]	Quadriceps Muscle Strength Correlates With Serum Vitamin D and Knee Pain in Knee Osteoarthritis	Vitamin D3	Significant correlation of quadriceps muscle strength with serum vitamin D and reduce knee pain by enhancing quadriceps strength in individuals with knee osteoarthritis (KOA).	**⇧**
Barker et al. (2014) [18]	Vitamin D deficiency associates with γ‐tocopherol and quadriceps weakness but not inflammatory cytokines in subjects with knee osteoarthritis	Observational, knee OA	Vitamin D deficiency negatively impacts quadriceps muscle function.	**⇧**
Heidari et al. (2015) [60]	Restorative Effect of Vitamin D Deficiency on Knee Pain and Quadriceps Muscle Strength in Knee Osteoarthritis	50,000 IU/week vitamin D3, 3 months	The correction of vitamin D deficiency has been shown to substantially improve quadriceps muscle strength and alleviate knee pain.	**⇧**
Wyon et al. (2014) [61]	The influence of winter vitamin D supplementation on muscle function and injury occurrence in elite ballet dancers: A controlled study	2000 IU/day vitamin D3, 4 months	Supplementing with oral vitamin D during winter has been associated with improved muscle function and strength a reduced risk of injury in ballet dancers.	**⇧**
Bruce et al. (2014) [62]	Vitamin D concentration in 342 professional football players and association with lower limb isokinetic function	25(OH)D different concentrations	Lower limb isokinetic peak torque does not consistently correlate with serum 25‐hydroxyvitamin D levels.	**⇔**
Owens et al. (2014) [63]	Vitamin D supplementation does not improve human skeletal muscle contractile properties in insufficient young males	4000 IU/day vitamin D3, 12 weeks	Raising serum total 25[OH]D levels above 120 nmol/L does not enhance muscle function, while impairment is observed only in cases of severe deficiency, with levels below 12.5 nmol/L.	**⇧**
Close et al. 2013 [64]	Assessment of vitamin D concentration in non‐supplemented professional athletes and healthy adults during the winter months in the UK: implications for skeletal muscle function	Non‐supplemented cohort	Inadequate vitamin D levels negatively affect musculoskeletal function in athletes.	**⇧**
Domingues‐Faria et al. (2014) [65]	Vitamin D deficiency down‐regulates Notch pathway contributing to skeletal muscle atrophy in old wistar rats	Vitamin d‐deficient diet	Vitamin D deficiency led to skeletal muscle atrophy by diminishing Notch signaling and cell proliferation, potentially worsening the natural decline in muscle regeneration associated with aging.	**⇧**

**Table 3:** Summary of Key Studies on Vitamin D and Functional Outcomes of human tendons.

Author, year, ref	Title	Intervention	Outcome	Effect
Thaveepunsan et al. (2024) [66]	Correlation Between Rotator Cuff Tear in Thai Urban Elderly Population and Vitamin D Deficiency	Observational, elderly	In an elderly urban Thai population, lower vitamin D levels were linked to larger tear size, greater fatty infiltration, reduced cartilage thickness, and more severe rotator cuff injuries.	**⇧**
Angeline et al. (2014) [67]	Effect of diet‐induced vitamin D deficiency on rotator cuff healing in a rat model	Vitamin d‐deficient diet	Insufficient vitamin D may negatively impact the initial healing phase following and reduced tensile strength rotator cuff repair.	**⇧**
Kim et al. (2020) [68]	A Combination Treatment of Raloxifene and Vitamin D Enhances Bone‐to‐Tendon Healing of the Rotator Cuff in a Rat Model	Vitamin D + raloxifene	Combined therapy with raloxifene (RLX) and vitamin D has been inhibiting further bone resorption and enhance tendon‐to‐bone healing and enhanced tensile strength in the human rotator cuff.	**⇧**
Min et al. (2019) [20]	Restoration of Cellular Proliferation and Characteristics of Human Tenocytes by Vitamin D	Vitamin D3, dose‐dependent	Increased mRNA levels, Collagen I synthesis, Restored tendon indices	**⇧**
Albright et al. (2024) [69]	Significant Association between a Diagnosis of Hypovitaminosis D and Rotator Cuff Tear, Independent of Age and Sex: A Retrospective Database Study	Observational, database	Vitamin D deficiency is a readily adjustable risk factor, and nutritional interventions have demonstrated beneficial effects on musculoskeletal health.	**⇧**

**Table 4:** Summary of Key Studies on Vitamin D and Functional Outcomes of human bones.

Author, year, ref	Title	Intervention	Outcome	Effect
Busa et al. (2023) [70]	Vitamin D reduces pain and cartilage destruction in knee osteoarthritis animals through inhibiting the matrix metalloprotease (MMPs) expression	Vitamin D3	Vitamin D exhibits anti‐inflammatory properties, reduces pain, and offers protective benefits to chondrocytes in rat models.	**⇧**
Sanghi et al. (2013) [71]	Does vitamin D improve osteoarthritis of the knee: a randomized controlled pilot trial	Vitamin D3	Vitamin D may offer modest improvements in pain relief and functional outcomes for individuals with knee osteoarthritis.	**⇧**
Yaka et al. (2022) [72]	Evaluation of the relationship between lateral epicondylitis and vitamin D	Vitamin D3, varying doses	Vitamin D deficiency could be a contributing factor in the development of lateral epicondylitis.	**⇧**
Ammerman et al. (2021) [73]	Prevalence of Vitamin D Insufficiency and Deficiency in Young, Female Patients with Lower Extremity Musculoskeletal Complaints	Vitamin D3	Vitamin D deficiency is common among women with musculoskeletal issues, particularly in cases involving ligament or cartilage damage	**⇧**
Wakefield et al. (2019) [74]	Bone structure is largely unchanged in growing male CD‐1 mice fed lower levels of vitamin D and calcium than in the AIN‐93G diet	Vitamin D3	No difference in femur midpoint or femur neck strength among the dietary intervention groups	**⇔**
Maroon et al. (2015) [22]	Vitamin D profile in National Football League players	Observational, NFL players	Athletes with insufficient vitamin D levels face an increased risk of sustaining bone fractures.	**⇧**
Hamson et al. (2014) [75]	Comparative study of bone mineral density, calcium, and vitamin D status in the Gujarati and white populations of Leicester	Vitamin D and BMD	Low serum vitamin D levels are prevalent among Gujaratis and highlights the need for additional research in South Asians to establish normal bone mineral density (BMD) values. Developing these reference ranges could improve the identification of osteoporosis risk in this population and carry important public health implications for the group.	**⇧**
Nicola et al. (2010) [76]	Vitamin D status and markers of bone turnover in Caucasian and South Asian postmenopausal women living in the UK	Vitamin D	The South Asian women had significantly lower 25(OH)D level than Caucasian women attending and reduced bone quality measured.	**⇧**
Allison et al. (2015) [77]	No association between vitamin D deficiency and markers of bone health in athletes	Observational, athletes	No association between 25[OH]D and BMD	**⇔**
Marian et al. (2008) [78]	Serum 25‐hydroxyvitamin D and bone mineral density in a racially and ethnically diverse group of men	Vitamin D	Highlights notable racial and ethnic disparities in both bone mineral density (BMD) and serum 25(OH)D levels among men, with a significant link between the two observed only in white men.	**⇧**
Heike et al. (2008) [79]	Dietary Calcium and Serum 25‐Hydroxyvitamin D Status in Relation to BMD Among U.S. Adults	Vitamin D	Improving 25‐hydroxyvitamin D levels may have a greater impact on bone mineral density than calcium supplementation alone.	**⇧**
Gerdhem et al. (2005) [80]	Association between 25‐hydroxy vitamin D levels, physical activity, muscle strength and fractures in the prospective population‐based OPRA Study of Elderly Women	Vitamin D	Association of decreased 25‐hydroxyvitamin D [25(OH)D] concentrations with increased incidence of fall‐related outcomes and increased fracture risk	**⇧**
Richard et al. (2009) [81]	Vitamin D status and its relationship to body fat, final height, and peak bone mass in young women	Vitamin D	Vitamin D has no role in maximum bone mass	**⇔**
Wölfl et al. (2013) [82]	Time course of 25(OH)D3 vitamin D3 as well as PTH (parathyroid hormone) during fracture healing of patients with normal and low bone mineral density (BMD)	Observational, fracture patients	No notable difference was found between individuals with normal and low bone mineral density (BMD), indicating the need for further research to better understand fracture healing and to develop clearer guidelines for treatment and monitoring.	**⇔**
Hamilton et al. (2010) [83]	Vitamin D deficiency is endemic in Middle Eastern sportsmen	Observational, sportsmen	25(OH)D deficiency is widespread among healthy male athletes in the Middle East, and due to its possible short‐ and long‐term health impacts, routine screening of serum 25(OH)D levels is recommended in this population.	**⇧**
Rabon‐Stith et al. (2005) [84]	Vitamin D receptor FokI genotype influences bone mineral density response to strength training, but not aerobic training	Strength training + Vitamin D	VDR FokI genotype may influence femoral neck bone mineral density response to strength training, but not aerobic exercise training.	**⇧**

Because included studies used heterogeneous endpoints, outcomes were grouped into consistent domains to improve readability and comparability: (1) **Strength** (e.g., isometric/isokinetic force, grip strength); (2) **Power/jumping performance** (e.g., vertical jump, sprint power); (3) **Balance/functional performance** (e.g., postural sway, gait, functional tests, falls); (4) **Biomechanics** (intrinsic/structural properties such as stiffness, modulus, load to failure, torque‐angle characteristics); and (5) **Injury risk/clinical outcomes** (e.g., injury incidence, pain, return‐to‐sport/return‐to‐function, fracture/tendon healing outcomes). Findings are presented within prespecified subgroups (children vs adolescents; athletes vs general/clinical/post‐operative cohorts) and are weighted toward higher‐quality trials.

**Skeletal Muscles:** Vitamin D supplementation enhanced quadriceps strength post‐ACL reconstruction [42] and mitigated weakness in knee osteoarthritis [60]. However, effects in young athletes were mixed, with some studies showing no strength gains [63]. Functional emphasis; sparse direct biomechanical endpoints in humans. Findings were mixed or weak, with several trials and observational analyses showing no improvement in muscle strength or performance in vitamin D–replete, trained individuals; where benefits were reported, they were inconsistent and often limited to subgroups with deficiency. Summary of Key Studies on Vitamin D and Functional Outcomes of human skeletal muscles is presented in Table 2.

**Tendons:** Preclinical and observational studies indicate that vitamin D may enhance tendon healing by promoting collagen synthesis [85] and reduced oxidative stress, suggesting therapeutic potential for tendinopathy [20], but robust human RCTs are needed to confirm these effects. More than 80% of patients undergoing rotator cuff repair exhibited vitamin D deficiency; however, the severity of rotator cuff tears showed no correlation with vitamin D levels [86]. Preclinical human translation uncertain; biomechanical endpoints lacking. Summary of Key Studies on Vitamin D and Functional Outcomes of human Tendons is presented in Table 3.

**Bones:** Observational studies suggest an association between vitamin D supplementation and a 15%–20% reduction in hip fracture risk in the elderly [87] and improved bone mineral density (BMD) post‐surgery [88], though causality cannot be confirmed without further RCTs. No consistent association between serum 25[OH]D and BMD were found in athletes [77]. Clinical outcomes predominate; biomechanical effects inconsistent in replete adults. Summary of Key Studies on Vitamin D and Functional Outcomes of human Bones is presented in Table 4.

Quality assessments (Table 5) confirmed high methodological rigor (PEDro scores 7–10).

**Table 5:** PEDro scales for article assessment.

No.	Author (year) (ref)	Eligibility criteria	Random allocation	Concealed allocation	Similarity at baseline	Patient blinding	Therapist blinding	Assessor blinding	Follow up > 85%	Intention to treat	Point and measures of variability	Between‐group statistical comparison	PEDro score	Quality level
1	Rebolledo et al. (2018) [40]	+	+	+	+	−	−	−	+	+	+	+	8	High
2	Bauer et al. (2019) [41]	+	+	+	+	−	−	−	−	+	+	+	8	High
3	Ong et al. (2024) [42]	+	+	+	+	+	−	−	+	+	+	+	9	High
4	Żebrowska et al. (2020) [43]	+	+	+	+	+	+	+	+	−	+	+	10	High
5	Ceglia et al. (2013) [44]	+	+	+	+	+	+	−	+	−	+	+	9	High
6	Visser et al. (2003) [45]	+	+	+	+	−	−	−	+	−	+	+	7	High
7	Bischoff et al. (2003) [46]	+	+	+	+	+	+	−	+	+	+	+	10	High
8	Dhesi et al. (2004) [17]	+	+	+	+	+	+	−	+	+	+	+	10	High
9	Pfeifer et al. (2009) [47]	+	+	+	+	+	+	−	+	+	+	+	10	High
10	Ceglia et al. (2010) [48]	+	+	+	−	−	−	−	+	−	+	+	6	Medium
11	Magalhães et al. (2024) [49]	+	−	+	+	−	−	−	+	+	+	+	7	High
12	Chen et al. (2022) [50]	+	−	−	+	−	−	+	+	+	+	+	7	High
13	Lee et al. (2021) [51]	+	−	+	+	−	−	−	+	+	+	+	7	High
14	Harada et al. (2019) [52]	+	−	+	+	−	−	−	+	+	+	+	7	High
15	Oh et al. (2009) [53]	+	−	+	+	−	−	−	+	+	+	+	7	High
16	Cancienne et al. (2019) [54]	+	−	+	+	−	−	−	+	+	+	+	7	High
17	Borges et al. (2020) [55]	+	−	+	+	−	−	−	+	+	+	+	7	High
18	Geusens et al. (1997) [56]	+	−	+	+	−	−	−	+	−	+	+	6	Medium
19	De Rezende et al. (2020) [57]	+	—	+	+	−	−	−	+	−	+	+	6	Medium
20	Dechsupa et al. (2023) [58]	+	−	+	+	−	−	−	+	+	+	+	7	High
21	Javadian et al. (2017) [59]	+	−	+	+	−	−	−	+	+	+	+	7	High
22	Barker et al. (2014) [18]	+	−	+	+	−	−	−	+	+	+	+	7	High
23	Heidari et al. (2015) [60]	+	−	+	+	−	−	−	+	+	+	+	7	High
24	Wyon et al. (2014) [61]	+	−	+	+	−	−	−	+	+	+	+	7	High
25	Bruce et al. (2014) [62]	+	−	+	+	−	−	−	+	+	+	+	7	High
26	Owens et al. (2014) [63]	+	+	+	+	−	−	−	+	+	+	+	8	High
27	Close et al. 2013 [64]	+	−	+	+	−	−	−	+	+	+	+	7	High
28	Domingues‐Faria et al. (2014) [65]	+	+	+	−	−	−	−	+	+	+	+	7	High
29	Thaveepunsan et al. (2024) [66]	+	−	+	+	−	−	+	+	+	+	+	8	High
30	Angeline et al. (2014) [67]	+	+	+	+	−	−	−	+	−	+	+	7	High
31	Kim et al. (2020) [68]	+	−	+	+	−	−	−	+	+	+	+	7	High
32	Min et al. (2019) [20]	+	−	+	+	−	−	−	+	+	+	+	7	High
33	Albright et al. (2024) [69]	+	+	+	+	−	−	−	+	+	+	+	8	High
34	Busa et al. (2023) [70]	+	+	+	+	−	−	−	+	−	+	+	7	High
35	Sanghi et al. (2013) [71]	+	+	+	+	+	+	+	+	+	+	+	11	High
36	Yaka et al. (2022) [72]	+	−	+	+	−	−	−	+	+	+	+	7	High
37	Ammerman et al. (2021) [73]	+	−	+	+	−	−	−	+	+	+	+	7	High
38	Wakefield et al. (2019) [74]	+	+	+	+	−	−	−	+	−	+	+	7	High
39	Maroon et al. (2015) [22]	+	+	+	+	−	−	−	+	+	+	+	8	High
40	Hamson et al. (2014) [75]	+	−	+	+	−	−	−	+	+	+	+	7	High
41	Nicola et al. (2010) [76]	+	−	+	−	−	−	−	+	+	+	+	6	Medium
42	Allison et al. (2015) [77]	+	−	+	−	−	−	+	+	+	+	+	7	High
43	Marian et al. (2008) [78]	+	+	+	+	−	−	−	+	+	+	+	8	High
44	Heike et al. (2008) [79]	+	−	+	−	−	−	+	+	+	+	+	7	High
45	Gerdhem et al. (2005) [80]	+	+	+	+	−	−	−	+	+	+	+	8	High
46	Richard et al. (2009) [81]	+	−	+	+	−	−	−	+	+	+	+	7	High
47	Wölfl et al. (2013) [82]	+	−	+	+	−	−	−	+	+	+	+	7	High
48	Hamilton et al. (2010) [83]	+	−	+	+	−	−	−	+	+	+	+	7	High
49	Rabon‐Stith et al. (2005) [84]	+	−	+	+	−	−	−	+	+	+	+	7	High

*Note:* The sign “‐” represents the absence of conditions and the sign “+” represents the presence of conditions. 1. Eligibility criteria were specified. 2. Subjects were randomly allocated to groups (in a crossover study, subjects were randomly allocated an order in which treatments were received). 3. Allocation was concealed.

### 4.1 Study Characteristics and Heterogeneity

Included studies varied substantially in participant age, baseline vitamin D status, 25(OH)D measurement and reporting, supplementation regimens, and outcome selection. We therefore documented assay/threshold reporting and synthesized results within prespecified age and population strata to minimize cross‐context extrapolation.

The groups were similar at baseline regarding the most important prognostic indicators.
There was blinding of all subjects.
There was blinding of all therapists who administered the therapy.
There was blinding of all assessors who measured at least one key outcome.
Measures of at least one key outcome were obtained from more than 85% of the subjects initially allocated to groups.
All subjects for whom outcome measures were available received the treatment or control condition as allocated or, where this was not the case, data for at least one key outcome was analysed by “intention to treat”.
The results of between‐group statistical comparisons are reported for at least one key outcome.
The study provides both point measures and measures of variability for at least one key outcome.

Criterion 1: This criterion is satisfied if the report describes the source of subjects and a list of criteria used to determine who was eligible to participate in the study.

Criterion 2: A study is considered to have used random allocation if the report states that allocation was random. The precise method of randomization need not be specified. Procedures such as coin‐tossing and dice‐rolling should be considered random. Quasi‐randomization allocation procedures such as allocation by hospital record number or birth date, or alternation, do not satisfy this criterion.

Criterion 3: Concealed allocation means that the person who determined if a subject was eligible for inclusion in the trial was unaware, when this decision was made, of which group the subject would be allocated to. A point is awarded for these criteria, even if it is not stated that allocation was concealed, when the report states that allocation was by sealed opaque envelopes or that allocation involved contacting the holder of the allocation schedule who was “off‐site”.

Criterion 4: At a minimum, in studies of therapeutic interventions, the report must describe at least one measure of the severity of the condition being treated and at least one (different) key outcome measure at baseline. The rater must be satisfied that the groups' outcomes would not be expected to differ, on the basis of baseline differences in prognostic variables alone, by a clinically significant amount. This criterion is satisfied even if only baseline data of study completers are presented.

Criteria 4, 7–11: Key outcomes are those outcomes which provide the primary measure of the effectiveness (or lack of effectiveness) of the therapy. In most studies, more than one variable is used as an outcome measure.

Criterion 5–7: Blinding means the person in question (subject, therapist or assessor) did not know which group the subject had been allocated to. In addition, subjects and therapists are only considered to be “blind” if it could be expected that they would have been unable to distinguish between the treatments applied to different groups. In trials in which key outcomes are self‐reported (eg, visual analogue scale, pain diary), the assessor is considered to be blind if the subject was blind.

Criterion 8: This criterion is only satisfied if the report explicitly states both the number of subjects initially allocated to groups and the number of subjects from whom key outcome measures were obtained. In trials in which outcomes are measured at several points in time, a key outcome must have been measured in more than 85% of subjects at one of those points in time.

Criterion 9: An intention to treat analysis means that, where subjects did not receive treatment (or the control condition) as allocated, and where measures of outcomes were available, the analysis was performed as if subjects received the treatment (or control condition) they were allocated to. This criterion is satisfied, even if there is no mention of analysis by intention to treat, if the report explicitly states that all subjects received treatment or control conditions as allocated.

Criterion 10: A between‐group statistical comparison involves statistical comparison of one group with another. Depending on the design of the study, this may involve comparison of two or more treatments, or comparison of treatment with a control condition. The analysis may be a simple comparison of outcomes measured after the treatment was administered, or a comparison of the change in one group with the change in another (when a factorial analysis of variance has been used to analyse the data, the latter is often reported as a group × time interaction). The comparison may be in the form hypothesis testing (which provides a “*p*” value, describing the probability that the groups differed only by chance) or in the form of an estimate (e.g., the mean or median difference, or a difference in proportions, or number needed to treat, or a relative risk or hazard ratio) and its confidence interval.

## 5 Discussion

This study aimed to review the impact of vitamin D consumption on the biomechanics of human tissues. We analyzed findings from various studies investigating how vitamin D affects the biomechanical properties of different tissues, including muscles, bones, and tendons. Relevant articles were identified through searches in specialized databases and selected based on predefined inclusion and exclusion criteria for further analysis. Importantly, observational associations between 25(OH)D concentrations and performance or injury outcomes do not establish therapeutic benefit. Throughout the Discussion, we distinguish correlational findings from supplementation trials and avoid therapeutic recommendations based solely on observational data. We also explicitly consider major confounders that influence both vitamin D status and musculoskeletal outcomes, including sun exposure, habitual physical activity (especially outdoor training), body composition/adiposity, and dietary protein and calcium intake. Contemporary large RCTs in broadly vitamin D–replete adults (e.g., VITAL and BMD ancillary analyses) show no reduction in fractures or improvement in BMD with vitamin D alone; therefore, we temper conclusions accordingly and align statements with GRADE certainty ratings (moderate‐to‐low for functional/falls in deficient older adults; very low for intrinsic biomechanical properties).

Vitamin D is a prohormone, regulates calcium and phosphate metabolism and steroid hormone calcitriol [12]. Vitamin D is a fat‐soluble vitamin that plays a crucial role in bone growth, skeletal health, and overall well‐being. Adequate levels of vitamin D help reduce the risk of low bone density, fractures, osteopenia, osteoporosis, and muscle weakness [13]. In addition, vitamin D has anti‐inflammatory effects on immune cells and plays a significant role in the pathogenesis and potential treatment of tendinopathy [14]. The physiologically active form of vitamin D, 1,25‐dihydroxyvitamin D3, reduces pro‐inflammatory cytokines such as interleukin‐6 (IL‐6), tumor necrosis factor‐alpha (TNF‐α), and interferon‐gamma (IFN‐γ), while increasing the anti‐inflammatory cytokine interleukin‐10 (IL‐10) in human peripheral blood mononuclear cells infected with Mycobacterium tuberculosis [15]. In addition, vitamin D regulates reactive oxygen species (ROS) levels, cyclooxygenase (COX) activity, and the nuclear factor kappa (NF‐κB) signaling pathway through its anti‐inflammatory effects [16]. Subsequently, we examined the impact of vitamin D intake on the biomechanical properties of various human tissues, including bones, muscles, and tendons.

### 5.1 Skeletal Muscles

Across included studies, evidence for a direct performance‐enhancing effect of vitamin D supplementation is strongest in populations with vitamin D deficiency and functional limitation (e.g., older adults or clinical groups), where some trials report improvements in balance, reaction time, or fall‐related outcomes. In contrast, in younger trained athletes the interventional evidence remains limited and inconsistent; therefore, observational links between low 25(OH)D and injury history should be interpreted as associations rather than proof of benefit from supplementation. Mechanistic and preclinical work suggests that vitamin D may influence muscle regeneration and tendon‐to‐bone healing pathways, but translation to measurable changes in intrinsic human tissue biomechanics has not been demonstrated consistently. Overall, vitamin D supplementation appears most relevant for correcting deficiency; routine supplementation to enhance performance or biomechanics in vitamin D–replete youth is not supported by current evidence.

Findings from various studies have investigated the effects of vitamin D consumption, with most confirming its beneficial impact on muscle function. Vitamin D deficiency is considered an easily modifiable risk factor, and several treatment regimens have demonstrated positive outcomes for musculoskeletal health [69]. Vitamin D supplementation has been shown to significantly improve performance, reaction time, and balance in individuals with a history of falls and vitamin D deficiency, though it does not appear to enhance muscle strength. These findings suggest that vitamin D may exert its effects through improved neuromuscular function or neuroprotective mechanisms, potentially explaining its role in reducing falls and fractures [17, 47]. Vitamin D levels are positively associated with muscle growth and metabolic function, suggesting a role in promoting muscle development and maintaining metabolic health [89]. Adequate or elevated levels of vitamin D in patients are associated with increases in the size, number, and strength of type II (fast‐twitch) muscle fibers [53, 89]. Given the high prevalence of falls among older adults, and the fact that type II muscle fibers are the first to be recruited in response to a fall, strengthening these fibers may play a crucial role in fall prevention in both elderly individuals and patients at risk [90]. This increase in muscle fiber proliferation may be linked to the Notch/BMP‐4 signaling pathway in myocytes. Although the exact mechanism remains unclear, reduced vitamin D levels have been associated with decreased expression of BMP‐4 on the surface of myocytes, potentially impairing muscle cell growth and proliferation [65]. Vitamin D plays a critical role in preventing musculoskeletal injuries in athletes, particularly following exercises involving abnormal muscle contractions [43]. Biomechanical and histological evidence indicates that low vitamin D levels negatively affect the healing of rotator cuff repair sites [51, 52, 66]. Postoperative patients with insufficient serum vitamin D levels have been shown to exhibit reduced muscle strength, underscoring the association between vitamin D status and rotator cuff muscle function [50, 53, 54, 91]. Athletes with a history of lower extremity muscle strains and core muscle injuries have been found to exhibit a higher prevalence of vitamin D deficiency [40]. Vitamin D supplementation has been shown to enhance quadriceps strength following anterior cruciate ligament reconstruction (ACLR) surgery [42] and may play an important anti‐inflammatory role in conditions driven by inflammation [18]. In such cases, co‐supplementation with vitamin D and calcium appears to be more effective, promoting greater muscle mass as well as stronger and more stable bones [92]. Vitamin D supplementation enhances rotator cuff tendon healing by promoting collagen synthesis and reducing oxidative stress, as demonstrated in preclinical studies. Oh et al. [53] demonstrated that increasing vitamin D levels in patients reduced fatty degeneration in the supraspinatus and infraspinatus muscles while improving isokinetic muscle performance. Additionally, a negative correlation was observed between vitamin D levels and the severity of muscle tears, suggesting a potential role for vitamin D in enhancing muscle recovery and maintaining muscle health [53]. In patients with knee osteoarthritis, correcting vitamin D deficiency has been shown to improve quadriceps strength and reduce knee pain [59, 60]. However, the potential for vitamin D to exert measurable effects on skeletal muscle performance in young, trained athletes remains controversial. The currently available evidence is limited, inconsistent, and often conflicting—some studies report beneficial effects [61, 64], while others find no significant impact [63, 93]. Although reviews have addressed the role of vitamin D in athletic performance, they often overlook non‐athletic populations. Given that athletes typically operate near their physiological limits, the scope for observable improvement is minimal, and any measurable effects are likely to be relevant only to peak performance contexts [94]. The broader claim that vitamin D supports muscle function is primarily supported by consistent findings in older adults. Several studies have reported increased proximal muscle strength in individuals with 25[OH]D concentrations below 25 nmol/L [95]. A review of sarcopenia in the elderly indicates that maintaining adequate vitamin D levels yields greater measurable benefits, and it is hypothesized that muscle function is primarily impaired by severe vitamin D deficiency a condition more prevalent in older populations [96]. Overall, the potential role of vitamin D in skeletal muscle regeneration following injury has been demonstrated Given its critical importance for skeletal muscle health, vitamin D levels should be routinely monitored and adequately maintained, particularly in elite young athletes [19].

Wang et al. Reported no VDR expression in skeletal muscle, challenging the role of VDR‐mediated pathways in vitamin D's effects on muscle function. This contrasts with Garcia et al., who found VDR‐mediated myogenic differentiation, suggesting variability may arise from differences in cell types or experimental conditions [97].

The role of vitamin D in muscle biomechanics, function, and healing has been extensively investigated, yet the evidence for its direct beneficial effects remains mixed, with several studies indicating limited or no significant impact on muscle recovery and performance.

Vitamin D is known to regulate muscle cell proliferation, differentiation, and calcium handling through its receptor (VDR) expressed in skeletal muscle fibers, suggesting a plausible mechanism for influencing muscle function and repair. It also modulates inflammatory responses and oxidative stress, which are critical in muscle damage and regeneration processes [98, 99]. Animal studies have demonstrated that vitamin D deficiency impairs muscle strength and regeneration, while supplementation can improve grip strength and endurance in mice. However, these positive findings in preclinical models do not consistently translate into clear functional benefits in humans [100].

Systematic reviews and meta‐analyses of human trials reveal that vitamin D supplementation does not reliably enhance muscle strength, function, or recovery after exercise‐induced muscle damage. For example, one systematic review concluded that despite vitamin D's immunomodulatory and antioxidant roles, supplementation failed to significantly reduce circulating muscle damage biomarkers or accelerate functional recovery following exercise [101]. Similarly, another review found inconsistent effects on muscle performance and recovery, with some studies showing no improvement or even negative effects on muscle strength and mobility, particularly with high‐dose supplementation [102].

The discrepancy between mechanistic insights and clinical outcomes may be attributed to several factors. First, vitamin D's influence on muscle may be indirect, primarily through improving bone health and neuromuscular coordination rather than directly enhancing muscle biomechanics. Second, baseline vitamin D status appears critical; benefits are more likely in individuals with severe deficiency, while supplementation in vitamin d‐replete subjects shows minimal or no effect [100]. Third, the optimal dosage, timing, and form of vitamin D for muscle healing remain unclear, with some evidence suggesting that excessive doses might impair muscle function by disrupting calcium homeostasis or mitochondrial activity [99].

Furthermore, human studies investigating vitamin D's effect on muscle healing after injury or surgery report mixed results. While some data indicate improved muscle regeneration and reduced inflammation with adequate vitamin D levels, other studies, such as those involving rotator cuff repair, show no significant benefit, highlighting the complexity of muscle repair mechanisms and the multifactorial nature of healing [100].

In conclusion, although vitamin D plays important roles in muscle cell biology and immune modulation, current clinical evidence does not robustly support a significant positive effect of vitamin D supplementation on muscle biomechanics, function, or healing in the general population. Its benefits may be limited to correcting deficiency states rather than enhancing muscle performance or recovery beyond normal physiological conditions. Future research should focus on well‐designed randomized controlled trials targeting deficient populations, standardized dosing regimens, and mechanistic studies to clarify vitamin D's precise role in muscle health [100, 101, 102].

### 5.2 Tendons

Research has explored how supplementing with vitamin D influences tendon health, with the majority of findings indicating beneficial outcomes. Evidence points to vitamin D playing a role in safeguarding damaged tenocytes, highlighting its potential as a treatment option for tendinopathy [20]. Vitamin D has a direct influence on the production of collagen by tenocytes. When human tendon fibroblasts were treated with vitamin D, researchers observed a dose‐responsive enhancement in type I collagen gene expression, accompanied by reduced oxidative stress and lower levels of matrix metalloproteinases. This suggests that vitamin D supports tendon health, and its absence may hinder collagen formation and elevate vulnerability to oxidative damage [85]. Population‐based studies have linked insufficient vitamin D levels with a higher risk of tendon disorders. One study found that 80% of individuals undergoing rotator cuff surgery were lacking in this nutrient [67, 86, 103]. Additionally, vitamin D was shown to promote tenocyte proliferation and restore tendon‐specific markers through activation of ERK and p38 signaling pathways. These findings point to its promise as a therapeutic agent in tendon healing through its regulation of immune function and cell growth [20]. Following surgical repair of the rotator cuff, the native four‐layered structure is lost, and new bridges composed of collagen fibers and extracellular matrix components begin to form. The regeneration of the tendon‐to‐bone interface is a multifaceted biological process, dependent on a range of elements such as local and recruited cells, signaling molecules like cytokines and growth factors, and structural proteins including different collagen types. Among these, collagen types I and III play essential roles. Type III collagen is predominant in the initial phase of healing and is commonly linked with scar formation and tissue degeneration, while type I collagen becomes more prevalent during the later remodeling phase [104]. Unsuccessful healing has been partially attributed to altered cartilage concentrations within the fibrovascular scar, where an imbalance in the transition from type III to type I collagen disrupts the necessary compositional ratio for proper tendon integration [67, 105]. Vitamin D contributes to enhancing the attachment of scar tissue to bone following surgery, thereby promoting more effective healing [67]. It exerts beneficial effects on rotator cuff tendons, in part by influencing the activity of matrix metalloproteinases (MMPs) [106]. Additionally, vitamin D plays a direct role in stimulating collagen production in tendon fibroblasts. When tendon cells derived from human tissue were treated with vitamin D2 or D3 metabolites, a dose‐dependent increase in type I collagen mRNA expression was observed, indicating an anabolic response [85]. Vitamin D helps lower intracellular levels of reactive oxygen species (ROS), offering protective benefits for tendon tissue. A deficiency in this nutrient can hinder the production of type I collagen and heighten oxidative stress in tendons. Beyond its antioxidant role, vitamin D also influences inflammatory pathways involved in tissue repair. For instance, in skin‐derived fibroblasts, it suppresses MMP1 expression, a mechanism that may contribute to higher tissue levels of type I collagen. Additionally, vitamin D3 has demonstrated anti‐inflammatory properties by reducing PGE2 production in IL‐1‐stimulated synovial fibroblasts [107]. In a rat model, rotator cuff healing was negatively affected by vitamin D deficiency induced through dietary restrictions and limited UV exposure [67]. Clinically, more than 80% of patients who underwent rotator cuff repair were found to have insufficient vitamin D levels. However, no direct association was identified between vitamin D concentration in the blood and tear size, severity, or the degree of fatty degeneration in the rotator cuff muscles. While vitamin D shows promise for supporting the repair of collagen‐rich tissues like tendons, especially following injury, robust clinical evidence remains limited [86] and promise in improving tendon strength and function, particularly in at‐risk populations such as athletes and the elderly [108] but its cost‐effectiveness requires evaluation through dedicated economic analyses.

The current body of evidence on the effects of vitamin D on tendon biomechanics, function, and healing reveals a nuanced and somewhat inconclusive picture, with several studies indicating limited or no direct beneficial impact. Although vitamin D is crucial for bone metabolism and muscle function, its role in tendon health is less clearly defined. Some animal and clinical studies suggest that vitamin D deficiency may negatively affect early tendon healing phases, particularly at the tendon‐to‐bone interface, by impairing bone mineral density and muscle strength that support tendon function. For example, diet‐induced vitamin D deficiency in animal models has been associated with compromised early rotator cuff healing, showing reduced biomechanical strength shortly after injury, although these differences tend to diminish over time, indicating no sustained long‐term biomechanical impairment. This suggests that vitamin D's influence may be more critical during initial healing stages but less so for ultimate tendon mechanical properties [67].

At the cellular level, vitamin D has demonstrated antioxidant and anti‐apoptotic effects in tenocytes, modulating inflammatory cytokines and promoting cell proliferation via pathways like ERK and p38 MAPK. However, these promising in vitro findings have not consistently translated into clear functional improvements in vivo. The variability in tendon injury models, dosing regimens, and outcome measures contributes to inconsistent results across studies [109].

Epidemiological data link vitamin D deficiency with increased incidence of tendinopathies and impaired healing, yet interventional trials with vitamin D supplementation often fail to show significant improvements in tendon strength, function, or healing outcomes, especially in populations without severe deficiency. This discrepancy suggests that while vitamin D status may be a marker of overall musculoskeletal health, supplementation alone may not directly enhance tendon biomechanics or repair [103, 108, 110].

In summary, despite mechanistic evidence supporting vitamin D's regulatory role in collagen synthesis, inflammation, and mineralization within tendons, current research does not conclusively support a direct positive effect of vitamin D supplementation on tendon biomechanics, function, or healing. The benefits of vitamin D appear largely mediated through improvements in bone quality and muscle strength rather than intrinsic tendon tissue properties. Future research should focus on well‐controlled clinical trials to determine if specific subpopulations, such as severely deficient individuals or athletes, might derive tendon‐specific benefits from vitamin D optimization.

### 5.3 Bones

Numerous studies have examined how vitamin D intake influences bone health, with most confirming its beneficial role. Maintaining sufficient levels of both calcium and vitamin D is crucial for minimizing the risk of fractures. In older adults, those with adequate levels of these nutrients experienced fewer hip fractures compared to individuals with vitamin D deficiency [87]. Hedström et al. [92] reported comparable findings in a study involving older women with hip fractures. Participants who were treated with a combination of vitamin D, calcium, and anabolic steroids demonstrated improved gait stability and functional performance at both 6‐ and 12‐months following surgery. However, the study did not clarify whether these improvements were directly attributable to vitamin D's influence on muscle recovery or primarily due to the effects of steroid therapy [87, 92]. Vitamin D levels serve as a key marker for calcium absorption efficiency and overall bone mineralization, helping to illustrate the connection between vitamin D deficiency and skeletal health [82, 111]. A variety of genetic, cultural, and environmental influences linked to low vitamin D status contribute to a higher likelihood of developing osteoporosis and experiencing fractures [17, 112, 113]. However, the existing research does not conclusively demonstrate a direct correlation between vitamin D deficiency and the degree of bone loss or fracture risk—especially among athletes, who are more prone to stress fractures [75, 76, 114, 115]. Recognizing the crucial roles that both calcium and vitamin D play in fracture healing can help clinicians make more informed treatment plans for their patients [21, 87, 88, 92]. As a dynamic tissue, bone responds to mechanical forces and possesses the capacity to repair and remodel itself [116]. Bone remodeling is a continuous and dynamic process involving three key stages: 1. resorption, where osteoclasts break down aged bone tissue; 2. reversal, during which mononuclear cells appear at the resorption site; and 3. formation, where osteoblasts construct new bone to replace the old [117, 118]. The activity of osteoblasts is closely tied to vitamin D function in the skeletal system. Vitamin D plays a regulatory role in bone remodeling by influencing both osteoblasts and osteocytes. Osteoblasts respond to various signals involved in bone turnover, such as the active form of vitamin D (1,25[OH]₂D₃) and parathyroid hormone (PTH). Through the induction of receptor activator of nuclear factor κB ligand (RANKL), vitamin D helps drive the remodeling process [119]. It also contributes to phosphate balance by stimulating fibroblast growth factor 23 (FGF23) [120] and may boost the bone's response to mechanical forces by activating mitogen‐activated protein kinase pathways [121]. Emerging evidence suggests that bone cells themselves are capable of converting 25[OH]D₃ into its active form, 1,25[OH]₂D₃, which may explain how circulating 25[OH]D₃ influences skeletal health [122]. Additionally, combining raloxifene with vitamin D has been shown to preserve bone mineral density (BMD) in animal models [68]. Gorter et al. [88] emphasized the crucial role of vitamin D in maintaining and restoring bone strength, both post‐surgery and during everyday physical activities, noting its contribution to elevated BMD. Higher BMD supports more effective bone healing [107, 108]. Physicians often use dual‐energy X‐ray absorptiometry scans to assess BMD, which aids in planning post‐operative recovery strategies [123]. Controlled laboratory studies—both cellular (in vitro) and animal‐based (in vivo)—are recommended to explore the specific effects of vitamin D on BMD at a microscopic level, enabling the development of more targeted medical treatments [103, 123]. Athletes, who experience significant mechanical stress during training and competitions, typically show increased BMD [124, 125]. This gain in body mass from physical training contributes to bone regeneration and the development of structurally robust bones [126]. Intense physical activity appears to stimulate the musculoskeletal system in a way that counteracts deficiencies in 25[OH]D, helping athletes avoid bone health issues despite low vitamin D levels [124, 127]. Athletes engaged in non‐weight‐bearing activities may experience similar detrimental skeletal effects [126, 128] and face a heightened risk of reduced BMD when deficient in vitamin D [129, 130]. However, recent studies have found no consistent correlation between serum 25[OH]D levels and bone integrity across athletic groups of diverse ethnic backgrounds, irrespective of exercise type (weight‐bearing vs. non‐weight‐bearing) [77]. These findings call into question the reliability of 25[OH]D levels as predictors of bone health in athletic populations. Genetic variations within the vitamin D metabolic pathway (25[OH]D/1,25[OH]2D) may partly account for this variability [83, 84]. Supporting this, emerging research has identified racial disparities in bone health biomarkers and their relationship to vitamin D levels [78, 94, 131]. For instance, Black football players have shown higher rates of vitamin D deficiency compared to their white counterparts, and those with insufficient levels face an increased likelihood of stress fractures [22]. The ideal concentration of serum 25[OH]D for skeletal well‐being remains uncertain. Many researchers suggest that adequacy should be defined by the level of 25[OH]D that either most effectively reduces parathyroid hormone (PTH) secretion or supports peak BMD [132, 133]. Studies on this topic have produced mixed results, especially among athletes and individuals from ethnic minority groups [79, 134]. More targeted research, particularly involving genetic analysis, is essential to clarify the direct effects of 1,25[OH]2D3 on osteoblast activity [78, 80, 81].

Current evidence on the effects of vitamin D on bone biomechanics, function, and healing presents a complex and somewhat conflicting picture, with numerous studies suggesting little or no beneficial effect—and in some cases potential negative outcomes—especially in vitamin D–replete populations.

Vitamin D is critical for calcium homeostasis and initial bone mineralization and deficiency is well‐known to cause secondary hyperparathyroidism, bone loss, osteomalacia, and increased fracture risk. However, research indicates that beyond preventing or correcting deficiency, additional vitamin D supplementation does not consistently improve biomechanical properties of bone or physical function. For example, Burt et al. (2019) conducted a 3‐year randomized clinical trial in healthy adults and found that supplementation with higher daily doses of vitamin D (4,000 IU and 10,000 IU) resulted in significantly lower radial and tibial bone mineral density compared with the standard 400 IU dose, with no improvement in bone strength measures observed. This suggests that excessive vitamin D intake may paradoxically diminish bone density and biomechanical integrity in individuals without deficiency [135].

Animal studies also accentuate this inconsistency. While some, such as a rat spinal fusion model, demonstrated improved femoral biomechanics and cortical thickness with increased dietary vitamin D, these findings are not universally replicated. In contrast, an ovariectomized rat model mimicking postmenopausal osteoporosis showed that long‐term treatment with calcium and vitamin D produced no effect on bone mineral density, microstructure, or biomechanical parameters, emphasizing the variability in response depending on the model and pathological context [136].

Regarding bone healing, vitamin D supplementation appears to have limited and inconsistent effects. Systematic reviews report no clear acceleration of fracture healing or significant improvements in functional recovery following supplementation in fracture patients without severe deficiency. Epidemiological data suggest potential fracture risk reduction in certain populations, mainly due to improved muscle function and decreased falls rather than direct mechanical strengthening of bone, but results remain inconclusive for vertebral and pediatric fractures [137, 138].

The lack or negativity of effect with vitamin D supplementation may arise from a threshold phenomenon: vitamin D status must be sufficient to maintain calcium absorption and basic bone homeostasis, but supraphysiological doses do not confer added biomechanical advantage and may disrupt bone remodeling balance. High intermittent or excessive daily dosing has been associated with increased bone resorption markers and decreased volumetric bone density, raising safety concerns [139, 140]. Moreover, sunlight exposure appears to have a more robust positive effect on bone structure and homeostasis than supplementation alone, further complicating the role of exogenous vitamin D [141].

In summary, despite its critical biological role in skeletal health, vitamin D supplementation, particularly in non‐deficient individuals, does not reliably enhance bone biomechanics, function, or fracture healing. High‐dose supplementation might even be detrimental to bone density and strength. Future clinical recommendations should focus on correcting deficiency states and avoiding unnecessary or excessive vitamin D dosing in healthy populations to prevent potential adverse skeletal effects [137, 138, 139, 140, 141].

Benefits observed in older/clinical cohorts—particularly when deficiency is corrected—should not be extrapolated to elite athletes, in whom effects on performance are inconsistent and frequently null. Baseline 25(OH)D status is a probable effect modifier, and training ceilings may limit detectable gains. Accordingly, we frame conclusions separately by population and emphasize deficiency correction over universal supplementation for performance.

### 5.4 Key Findings

This review is primarily function‐oriented and reports biomechanical evidence only when directly measured; intrinsic biomechanical effects in humans remain uncertain. Mechanistic studies suggest that vitamin D signaling may influence muscle regeneration pathways (e.g., Notch/BMP‐4) [65] and inflammatory responses [42]; however, human interventional evidence indicates that functional benefits are most consistently observed when deficiency is corrected in older or clinical populations [95], whereas effects on performance in athletes and vitamin D–replete youth are inconsistent [94]. For tendon and tendon‐to‐bone healing, supportive evidence is largely preclinical and observational, and cannot be interpreted as proof of improved intrinsic mechanics in humans [20, 86]. For bone outcomes, supplementation does not reliably improve biomechanical strength in vitamin D–replete individuals; potential benefits appear concentrated in deficient subgroups and depend on co‐factors such as calcium intake, training status, and overall health [65, 87].

### 5.5 Implications and Comparisons

Unlike Stockton et al. [95], which found consistent strength gains, our review notes mixed effects in athletes, suggesting context‐specific benefits. Vitamin D's anti‐inflammatory role (e.g., reducing IL‐6, TNF‐α) [15] may underpin its biomechanical effects, particularly in injury recovery. However, optimal serum 25[OH]D levels (e.g., 30–50 nmol/L) for functional outcomes (primary); biomechanical endpoints (secondary, when reported) remain debated [132] and findings from observational studies should be interpreted with caution due to potential confounding, and causal claims require validation through RCTs.

### 5.6 Study Quality

Overall, the internal validity of included RCTs was high (PEDro 7–10; Table 5). Domains most commonly satisfied related to randomization, baseline comparability, and complete reporting of between‐group differences with measures of variability. As expected for supplementation and rehabilitation studies, blinding of participants and therapists was infrequently achievable, which may increase risk of performance bias in some trials. Because study quality varied by design, we explicitly weighted our interpretation of findings: causal inferences were drawn primarily from high‐quality RCT evidence, while observational and mechanistic studies were used to describe associations and biological plausibility, and were not used to support strong causal claims. Conclusions are therefore framed by population subgroup and baseline vitamin D status, and claims are limited to the certainty supported by the highest‐quality evidence available. Accordingly, primary conclusions regarding supplementation effects are based on high‐quality RCTs (PEDro > = 7). Observational and mechanistic findings are presented as supportive context and hypothesis‐generating evidence rather than causal proof.

### 5.7 Limitations

Given substantial clinical and methodological heterogeneity (age ranges, 25(OH)D assays and reporting units, study‐specific deficiency thresholds, dosing regimens, and non‐comparable outcomes), statistical pooling was not appropriate. Instead, we implemented a predefined, evidence‐weighted stratified narrative synthesis by age group (children vs adolescents vs adult/older cohorts when included) and population context (athletes vs general/clinical vs post‐operative), and grouped outcomes into Functional/Performance, Clinical, and Biomechanical domains. Limitations include small sample sizes, variable dosages (400–4000 IU/day), heterogeneous outcome measures, and sparse athlete‐specific data, which limit generalizability. Combined interventions (e.g., with calcium or exercise) complicate isolating vitamin D's effects, though synergistic benefits are evident [87]. In addition, many observational studies are vulnerable to residual confounding because determinants of vitamin D status also influence musculoskeletal outcomes. Sun exposure and outdoor training can raise 25(OH)D and independently improve fitness and injury resilience; physical activity level and training load are strong predictors of performance; adiposity is inversely associated with circulating 25(OH)D and may affect biomechanics; and dietary calcium and protein intake can modify bone and muscle outcomes and may co‐vary with supplement use. Although several studies adjusted for some covariates, adjustment was inconsistent across the literature, limiting causal interpretation of associations.

### 5.8 Funding Source Review

Funding sources of included studies were reviewed, with no significant conflicts identified.

### 5.9 Recommendations for Future Research

Trials and well‐designed cohorts should also measure and/or control key confounders (sun exposure, habitual physical activity/training load, body composition, and dietary calcium/protein) to strengthen causal inference and improve comparability across studies.

### 5.10 Clinical Implications

The evidence summarized in this review supports a cautious, deficiency‐focused approach to clinical decision‐making. To strengthen transparency, we distinguish evidence‐supported implications from expert considerations where evidence is limited.

Evidence‐supported implications (based on the reviewed intervention and clinical‐outcome literature):

Screening thresholds (interpretation categories): Many studies and clinical frameworks interpret serum 25(OH)D concentrations using pragmatic categories such as deficiency < 50 nmol/L (< 20 ng/mL), insufficiency 50–75 nmol/L (20–30 ng/mL), and sufficiency > = 75 nmol/L (> = 30 ng/mL). Because included studies used heterogeneous cut‐offs and assays, clinicians should interpret values in context and, where possible, use consistent local laboratory standards.
When supplementation may be warranted: Across populations, the strongest justification for supplementation is documented deficiency or high likelihood of deficiency with relevant symptoms or risk factors. The interventional evidence summarized here does not support routine vitamin D supplementation solely to enhance athletic performance or tissue biomechanics in vitamin D–replete individuals. Rather, supplementation is most defensible as a strategy to correct deficiency, particularly in individuals with functional limitations, impaired recovery, or clinical contexts where low vitamin D is common.
Populations at increased risk: Youth and adolescents may be at higher risk of low vitamin D status in settings of limited sun exposure (high latitude, winter season, indoor training, cultural clothing practices, diligent sunscreen use), higher adiposity, darker skin pigmentation, dietary patterns low in vitamin D, and conditions affecting absorption or metabolism. In sports medicine settings, indoor athletes and those training predominantly outside peak UVB months may warrant closer attention.

### 5.11 Expert Considerations (Where Evidence Is Indirect, Heterogeneous, or Not Specific Enough for Universal Recommendations)

**Who to screen:** Universal screening is not addressed uniformly across the included studies; however, targeted screening may be reasonable for individuals with risk factors above, recurrent stress injuries, delayed recovery, or clinical features suggestive of deficiency.
**Co‐factors that may modify outcomes:** Interpretation and management should consider **calcium and protein intake**, **overall energy availability**, training load, and body composition, which may confound observational associations and modify response to supplementation.
**Implementation and safety:** Supplementation should follow prevailing clinical guidance regarding dosing, monitoring, and avoidance of excessive intake, particularly when high‐dose regimens are used.

Overall, the clinical message is that vitamin D status is relevant to musculoskeletal health, but the most evidence‐consistent application is **identifying and correcting deficiency**, rather than using supplementation as a general performance‐enhancing intervention.

## 6 Conclusion

This systematic review indicates that vitamin D status is plausibly related to musculoskeletal health and some functional outcomes; however, the certainty of evidence varies by population, baseline vitamin D status, and outcome type. Interventional evidence does not support routine vitamin D supplementation as a general performance enhancer in vitamin D–replete youth or athletes. Potential benefits appear most defensible in the context of correcting deficiency and in higher‐risk groups, while evidence for direct changes in intrinsic human tissue biomechanics remains limited. Future research should prioritize well‐powered RCTs in clearly defined pediatrics/adolescent cohorts with standardized 25(OH)D assays and reporting, prespecified outcome domains (strength, power, balance, biomechanics, injury outcomes), and careful measurement/control of key confounders (sun exposure, training load, body composition, and dietary protein/calcium). Clinical recommendations should prioritize deficiency identification and correction; where evidence is limited, we label implications as expert considerations rather than evidence‐based guidance.

## Author Contributions

**Mohammad Soltani:** conceptualization, methodology, writing – original draft, data curation, investigation, resources, writing – review and editing. **Ali Fatahi:** project administration, formal analysis, visualization, supervision, software, validation, writing – review and editing. **Fatemeh Heidari:** supervision, validation, writing – review and editing.

## Funding

The authors have nothing to report.

## Conflicts of Interest

The authors declare no conflicts of interest.

## Transparency Statement

The lead author Ali Fatahi affirms that this manuscript is an honest, accurate, and transparent account of the study being reported; that no important aspects of the study have been omitted; and that any discrepancies from the study as planned (and, if relevant, registered) have been explained.

## Data Availability

The data presented in this study are available on request from the corresponding author. The data that support the findings of this study are available from the corresponding author upon reasonable request.
